# Different Areas of Chronic Stress and Their Associations with Depression

**DOI:** 10.3390/ijerph19148773

**Published:** 2022-07-19

**Authors:** Felix S. Hussenoeder, Ines Conrad, Alexander Pabst, Melanie Luppa, Janine Stein, Christoph Engel, Silke Zachariae, Samira Zeynalova, Maryam Yahiaoui-Doktor, Heide Glaesmer, Andreas Hinz, Veronica Witte, Gunnar Wichmann, Toralf Kirsten, Markus Löffler, Arno Villringer, Steffi G. Riedel-Heller

**Affiliations:** 1 Institute of Social Medicine, Occupational Health and Public Health, Leipzig University, 04103 Leipzig, Germany; ines.conrad@medizin.uni-leipzig.de (I.C.); alexander.pabst@medizin.uni-leipzig.de (A.P.); melanie.luppa@medizin.uni-leipzig.de (M.L.); janine.stein@medizin.uni-leipzig.de (J.S.); steffi.riedel-heller@medizin.uni-leipzig.de (S.G.R.-H.); 2 Institute for Medical Informatics, Statistics and Epidemiology (IMISE), Leipzig University, 04107 Leipzig, Germany; christoph.engel@imise.uni-leipzig.de (C.E.); silke.zachariae@imise.uni-leipzig.de (S.Z.); samira.zeynalova@imise.uni-leipzig.de (S.Z.); maryam.yahiaoui-doktor@medizin.uni-leipzig.de (M.Y.-D.); toralf.kirsten@medizin.uni-leipzig.de (T.K.); markus.loeffler@imise.uni-leipzig.de (M.L.); 3 Leipzig Research Centre for Civilization Diseases, Leipzig University, 04103 Leipzig, Germany; 4 Department of Medical Psychology and Medical Sociology, Leipzig University, 04103 Leipzig, Germany; heide.glaesmer@medizin.uni-leipzig.de (H.G.); andreas.hinz@medizin.uni-leipzig.de (A.H.); 5 Max-Planck-Institute for Human Cognitive and Brain Sciences, 04303 Leipzig, Germany; witte@cbs.mpg.de (V.W.); arno.villringer@medizin.uni-leipzig.de (A.V.); 6 Department of Otorhinolaryngology, University of Leipzig, 04103 Leipzig, Germany; gunnar.wichmann@medizin.uni-leipzig.de; 7 Department for Medical Data Science, University Hospital Leipzig, 04103 Leipzig, Germany

**Keywords:** chronic stress, depression, TICS, CES-D, public mental health

## Abstract

Background: Research shows a connection between stress and depression, but there is little differentiation between areas of stress, making it difficult to identify and address specific areas in the context of public health measures. We utilized a multi-dimensional approach to chronic stress to better understand the relationship between different areas of stress and depression. Methods: We conducted linear regression analyses and used data from a sub-sample of the LIFE-Adult-Study (N = 1008) to analyze the connection between nine different areas of chronic stress (TICS) and depression (CES-D). In the second analysis, we controlled for sociodemographic variables, personality, physical activity, and social support. Results: There was a significant positive association between the stress domains Excessive Demands from Work, Lack of Social Recognition, Social Isolation, and Chronic Worrying and depression and a significant negative association between Pressure to Perform and depression. After adding control variables, only Pressure to Perform, Social Isolation, and Chronic Worrying remained significant predictors. Conclusions: By focusing on the connections between chronic stress and depression, researchers can help identify the areas that matter most and contribute to the creation of meaningful and efficient interventions. On the basis of our results, measures for the prevention of depression that focus on the reduction of worrying and social isolation are recommended.

## 1. Introduction

Research shows an empirical connection between stress and depression in a variety of different populations—from university students in Canada, Egypt, and Korea ([1,2,3] to farmers [4], physicians [5], and bank employees [6]—and throughout the life course, from high school students to elderly people [7,8]. While experimental studies especially point toward a causal effect of stress on depression [9,10,11], there is also empirical evidence indicating the opposite trend [12,13], and several studies have highlighted bidirectional, more complex, and even intergenerational relationships between depression and stress [14,15,16].

In the vast majority of studies, stress is treated as a one-dimensional and broad phenomenon, and there is no differentiation between domains of stress. This limits the ability of researchers to identify the areas of stress that are the most relevant to depression as well as the ability of policymakers and practitioners to develop meaningful and efficient public mental health measures based on that research. Chronic stress is a form of stress that is especially important when it comes to mental health, and research shows an empirical connection not only with depression but also with other disorders and symptoms, such as burnout and cognitive impairment [11,17]. Chronic stress is so detrimental because it represents a form of permanent arousal that, unlike more acute forms of stress, prevents mechanisms of relaxation and homeostasis [18]. In addition, chronic stress is linked to changes in brain physiology and functioning [19,20,21].

In order to better understand the relationships between different domains of chronic stress and depression, we used the approach used by Schulz et al. [22], which focused on nine different stress domains measured by the Trier Inventory for Chronic Stress (TICS): (1) Work Overload, (2) Social Overload, (3) Pressure to Perform, (4) Work Discontent, (5) Excessive Demands from Work, (6) Lack of Social Recognition, (7) Social Tensions, (8) Social Isolation, and (9) Chronic Worrying. Studies have connected TICS-measured chronic stress to depression; however, they have either only used parts of the instrument [23] or a short version that does not differentiate between areas of stress [24,25]. Therefore, in this study, we aimed to fill a gap in the literature by analyzing the connection between all nine areas of chronic stress and depression with data from a German cohort study. Our goal was to identify the areas of stress that are most relevant to depression and thereby provide the basis for further scientific study as well as future public mental health measures.

## 2. Methods

### 2.1. Study Design

The Adult Study of the Leipzig Research Centre for Civilization Diseases (LIFE) was a large population-based study in the city of Leipzig, Germany, and a unique collaboration between several clinical and epidemiological research teams. Between 2011 and 2014, participants between 18 and 80 years of age were recruited through age- and gender-stratified random selection by the local residents’ registry office, with pregnancy being the only exclusion criterion. Every participant provided written informed consent prior to participation, and participants underwent a set of assessments, including interviews, questionnaires, and medical examinations. The details of the study design and assessments can be found elsewhere [26]. For our analysis, we used a subsample of participants that filled out the TICS questionnaire. The final sample for analysis contained 1008 participants.

### 2.2. Ethics

The LIFE-Adult-Study complied with the ethical standards of the relevant national and institutional committees on human experimentation and with the Helsinki Declaration of 1975, as revised in 2008. The study was approved by the ethics committee of Leipzig University.

### 2.3. Measures

#### 2.3.1. Sociodemographic Variables

Participants were asked for information on age, gender, and marital status in standardized interviews by trained study personnel. They also provided details on occupational status, education, and equivalent household income that were used to compute their socioeconomic status [27].

#### 2.3.2. Stress

We used the Trier Inventory for Chronic Stress (TICS) [22] to assess nine interrelated areas of chronic stress via 57 items: (1) Work Overload (e.g., “I have too many tasks to perform.”), (2) Social Overload (e.g., “I must frequently care for the well-being of others.”), (3) Pressure to Perform (e.g., “I have tasks to fulfill that pressure me to prove myself.”), (4) Work Discontent (e.g., “Times when none of my tasks seem meaningful to me.”), (5) Excessive Demands from Work (e.g., “Although I try, I do not fulfill my duties as I should.”), (6) Lack of Social Recognition (e.g., “Although I do my best, my work is not appreciated.”), (7) Social Tensions (e.g., “I have unnecessary conflicts with others.”), (8) Social Isolation (e.g., “Times when I have too little contact with other people.”), and (9) Chronic Worrying (e.g., “Times when I worry a lot and cannot stop.)” (item translation from Petrowski et al., 2012 [28]). Participants were asked to rate how often they had experienced a specific situation or had a specific experience within the last three months on a Likert scale from 0 (“=never”) to 4 (“very often”). Higher scores indicated higher levels of stress. The TICS provides good psychometric properties [28].

#### 2.3.3. Depression

We used the German version of the Center for Epidemiologic Studies Depression Scale (CES-D Scale) [29,30]. The scale contains 20 items addressing the frequency of depressive symptoms, such as depressed mood, hopelessness, poor appetite, and feelings of guilt, during the past week on a scale from 0 (rarely/none of the time) to 3 (most of the time). The total score ranged between 0 and 60, and higher scores represented increased levels of depression. The scale is well-established, and it provides good psychometric properties [30].

#### 2.3.4. Covariates

Participants answered the revised German version of the Ten Item Personality Inventory (TIPI) [31], which comprises 16 items (scale range: 1–7). It is used to assess the Big Five personality traits, i.e., Neuroticism, Extraversion, Openness to Experience, Agreeableness, and Conscientiousness. We assessed the level of social support via the 5-item ENRICHD Social Support Scale [32]. We included personality and social support as control variables because a meta-analysis suggested their relationship with depression [33,34].

### 2.4. Statistical Analysis

Statistical analyses were performed usingSPSS 25.0 (IBM Corp., Armonk, NY, USA). We used a subset of the original LIFE-Adult cohort, comprising the subjects who filled out the TICS questionnaire. Since the TICS assesses chronic stress over a period of three months, we excluded all participants from the analysis for whom depression had been assessed more than 90 days before stress (N = 100). We then removed subjects with missing values for any of the predictors, control variables, or the outcome variable (N = 463). The final sample contained 1008 participants.

We conducted a correlational analysis (Pearson’s correlation, two-tailed) and a linear regression analysis with all areas of chronic stress as predictors (Work Overload, Social Overload, Pressure to Perform, Work Discontent, Excessive Demands from Work, Lack of Social Recognition, Social Tensions, Social Isolation, or Chronic Worrying) and depression as the outcome. For the second regression analysis, we added age, gender, socioeconomic status, marital status, personality, and social support as control variables.

## 3. Results

Our sample contained 1008 participants; 43.3% were females, and the mean age was 54.4 years. Table 1 gives an overview of the general characteristics of our sample.

Table 2 shows that all areas of stress were significantly and positively correlated with each other and also with depression, with the only exception being Pressure to Perform. When we used the well-established cutoffs 0.1, 0.3, and 0.5 [35] to categorize the correlations between stress domains and depression, there were small effects for Work Overload, Social Overload, Lack of Social Recognition, and Social Tensions, a medium effect of Work Discontent, Excessive Demands from Work, and Social Isolation, and a large effect of Chronic Worrying.

Table 3 shows a significant negative association between Pressure to Perform and depression and significant positive associations between Excessive Demands from Work, Lack of Social Recognition, Social Isolation, and Chronic Worrying and depression. After adding control variables, only the negative association between Pressure to Perform and depression and the positive associations between Social Isolation and Chronic Worrying and depression stayed significant. There was a significant positive association between age, Neuroticism, and depression, and a significant negative one between high socioeconomic status, social support, and depression.

## 4. Discussion

The correlational analysis showed connections between all domains of chronic stress and depression besides Pressure to Perform, while the multivariate analysis exhibited a significant positive association between Excessive Demands from Work, Lack of Social Recognition, Social Isolation, and Chronic Worrying and depression and a significant negative association between Pressure to Perform and depression. After adding control variables, only the associations with Pressure to Perform, Social Isolation, and Chronic Worrying remained significant.

Our results showed the strongest connection between stress and depression with regard to Chronic Worrying, an association that is supported by other studies [36,37,38]. This connection could, to some extent, be the consequence of a common underlying factor; the literature suggests that Neuroticism predicts chronic stress and worry [39,40] as well as depression [41,42] and that Neuroticism may, to some extent, account for the connection between depression and chronic stress [43]. While the cross-sectional nature of our study does not lend itself to causal interpretations, the fact that Neuroticism significantly predicted depression and the medium-sized correlation between Neuroticism and Chronic Worrying (0.43, *p* ≤ 0.001; not displayed in the results) are in line with the literature. These results are also compatible with studies that suggest that worrying may act as a mediating variable between Neuroticism and depression [44]. With the role of worrying as a predictor and/or mediator that is directly linked to depression, interventions that target worrying—such as cognitive behavioral and mindfulness-based interventions [45,46]—could be an efficient and easily applicable way to mitigate stress and prevent depression. In addition, while personality traits are rather stable, a review by Roberts et al. [47] showed that especially Neuroticism may change as a consequence of interventions.

Multiple areas of the TICS cover social relationships, but in the final regression, only Social Isolation remained a significant predictor of depression. The relevance of social isolation for depression is reflected in the literature [48,49] as are the protective effects of perceived support, and social networks [50]. Since longitudinal research suggests that social isolation can cause depression [51], addressing social isolation could help mitigate depression. In line with this argument, studies have shown that facilitating social group memberships [52,53] and promoting peer support [54,55] could be promising avenues for reducing depressive symptoms. Our results suggest that the main focus of interventions should be on reducing social isolation and that chronic stress from social interaction—in terms of social overload, lack of recognition, and social tensions—is less important.

There was a significant negative association between Pressure to Perform and depression, which initially seems counterintuitive. However, this result is most likely a consequence of the fact that Pressure to Perform refers to performing tasks and behaviors that are evaluated and judged by others and, therefore, involves a strong “social component”. Typical items address “situations where I have to make an effort to win the trust of others” or “tasks that involve being critically evaluated” [22]. The negative association between this specific domain of chronic stress and depression could thus be a consequence of the withdrawal from social situations and social isolation, which are associated with depression and are also reflected in our data. In addition, since pressure to perform is also closely linked to the working environment, these results match with studies that suggest a connection between reduced work engagement, as well as absenteeism and presentism at work and depression [56,57,58]. They are also in line with our other results, which indicated that work-related domains of chronic stress only play a marginal role in depression. While Excessive Demands from Work mainly refers to the complexity of tasks, Work Discontent is concerned with the qualitative aspects of tasks (e.g., the fact that they are perceived as meaningless, uninteresting, and not matching with one’s skills) and Work Overload is concerned with the number of tasks. None of the three domains was significantly associated with depression in the final regression.

## 5. Limitations

While our study has several strengths, such as the large sample size, the multi-faceted understanding of chronic stress, and the inclusion of personality traits as covariates, it also has some limitations. First, future research would benefit from a longitudinal design to further clarify the direction of causality on the level of single areas of chronic stress. Second, we applied a multi-faceted perspective on stress but a one-dimensional one on depression. Future research should consider using a more detailed approach to depression to analyze whether different areas of stress are associated with different elements of depression.

## 6. Conclusions

Our study showed significant associations between specific domains of chronic stress and depression. It thereby contributes to the identification of those areas of stress that are important for future mental health research and the development of public health measures and interventions. Our results suggest that future measures should focus on the reduction of worrying and social isolation, and future research could evaluate the effects of those measures.

## Figures and Tables

**Table 1:** General characteristics of the study population.

	Total Group (N = 1008)
Age (years)	54.4 (16.8)
Gender (“female”)	436 (43.3%)
Socioeconomic status ^1^	
Low	151 (15.0%)
Medium	589 (58.4%)
High	268 (26.6%)
Marital status	
Married	579 (57.4%)
Single	298 (29.6%)
Divorced	85 (8.4%)
Widowed	46 (4.6%)
Personality (TIPI; range: 1–7)	
Neuroticism	3.2 (1.1)
Extraversion	3.6 (1.2)
Openness	5.5 (0.9)
Agreeableness	5.8 (0.9)
Conscientiousness	5.8 (0.9)
Social support (ESSI; range: 5–25)	22.4 (3.3)
Stress (TICS; range: 0–4)	
Work Overload	1.2 (0.8)
Social Overload	1.4 (0.8)
Pressure to Perform	1.5 (0.8)
Work Discontent	1.1 (0.6)
Excessive Demands from Work	0.8 (0.6)
Lack of Social Recognition	1.1 (0.7)
Social Tensions	0.9 (0.6)
Social Isolation	1.0 (0.7)
Chronic Worrying	1.2 (0.8)
Depression (CES-D; 0–60)	9.6 (6.2)

Note: Continuous variables are given as means (standard deviation); categorical variables are displayed as numbers (percentages). ^1^ Socioeconomic status was computed on the basis of education, occupational status, and equivalent household income [27].

**Table 2:** Correlations between areas of chronic stress and depression (N = 1008).

	1	2	3	4	5	6	7	8	9	10
1. Work Overload	1									
2. Social Overload	0.64 ***	1								
3. Pressure to Perform	0.66 ***	0.69 ***	1							
4. Work Discontent	0.31 ***	0.21 ***	0.33 ***	1						
5. Excessive Demands from Work	0.53 ***	0.32 ***	0.39 ***	0.57 ***	1					
6. Lack of Social Recognition	0.50 ***	0.41 ***	0.46 ***	0.51 ***	0.55 ***	1				
7. Social Tensions	0.39 ***	0.43 ***	0.43 ***	0.40 ***	0.44 ***	0.48 ***	1			
8. Social Isolation	0.21 ***	0.18 ***	0.24 ***	0.56 ***	0.41 ***	0.38 ***	0.32 ***	1		
9. Chronic Worrying	0.46 ***	0.34 ***	0.33 ***	0.49 ***	0.61 ***	0.41 ***	0.44 ***	0.47 ***	1	
10. Depression	0.19 ***	0.11 ***	0.04	0.35 ***	0.40 ***	0.29 ***	0.27 ***	0.43 ***	0.53 ***	1

Note: Pearson’s correlation, two-tailed; *** *p* ≤ 0.001.

**Table 3:** Prediction of depression by areas of chronic stress with and without control variables (standardized regression coefficients; N = 1008).

	Depression	
Domains of Chronic Stress		
Work Overload	0.01	0.06
Social Overload	0.04	0.03
Pressure to Perform	−0.26 ***	−0.16 ***
Work Discontent	0.00	0.03
Excessive Demands from Work	0.10 **	0.06
Lack of Social Recognition	0.07 *	0.04
Social Tensions	0.04	0.02
Social Isolation	0.22 ***	0.15 ***
Chronic Worrying	0.40 ***	0.31 ***
Age		0.10 **
Gender (female) ^1^		−0.00
Socioeconomic status (low)		
medium		−0.02
high		−0.08 *
Marital status (married) ^2^		0.04
Personality		
Neuroticism		0.16 ***
Extraversion		0.03
Openness		−0.05
Agreeableness		0.03
Conscientiousness		−0.00
Social support		−0.13 ***
R^2^	0.37	0.42

Note: * *p* ≤ 0.05; ** *p* ≤ 0.01; *** *p* ≤ 0.001. ^1^ The category coded as “0” (=reference category) is presented in parentheses. ^2^ Marital status: 0 = married; 1 = not married.

## Data Availability

The data that support the findings of this study are available from the corresponding author upon reasonable request.
